# 

**Published:** 1858-10

**Authors:** 


### NOTES AND CORRESPONDENCE.

The Editor is anxious to give free scope to the expression of opinion, but will not hold himself responsible for the opinions of those of his contributors who attach their names to the papers.

It gives us great pleasure to state that the honour of C.B. has been conferred on Dr. McWilliam, the hero of the famous Niger Expedition and the indefatigable and learned Secretary of the Epidemiological Society. Honours of this kind are often given out of mere compliment. It, therefore, does honour to the honour when the presentation is in recognition of simple merit, as is the fact in the present instance. There are men in medicine who get promotions and dignities of which everybody knows and nobody cares. But when Dr. McWilliam gets an honour everybody is glad, and all wonder why the honour was so long reserved! There is a clear reason for this general sympathy: it is reflex. Dr. McWilliam is the generous friend of all who know him.

By order of a Treasury minute, the Returns of the Registrar General are no longer supplied to the scientific world by favour. Even from this Review, the only special literary representative, in our common language, of sanitary science, the returns are withheld. We must buy the information which, with all our contributors, we assist in supplying. This new rule is, without exception, one of the most undignified acts ever perpetrated by any body of officials extant or ever extant. When the reason is asked—Why this withholding? the answer given is, That the Treasury Lords intend a great national saving of money, by selling knowledge, instead of giving it away. In a word, the Treasury Lords have opened a publishing business, from which they will realise for the nation say two thousand a year, the nation itself having previously advanced the money. If the question be asked—How is the contemptible spectacle of a Treasury Lords publishing shop to be shut up? the answer is supplied by another question—How can books be published if there are no authors? Let only those liberal Authors of the Government publishers, who supply, for example, meteorological reports, reports of death causes, and the like, take a holiday. This is the recipe! Nothing more is required.

### BOOKS RECEIVED.

An Act to Alter and Amend the Metropolis Local Management Act. 1858.

An Act to Regulate the Qualifications of Practitioners in Medicine and Surgery. 1858.

An Act to Amend the Public Health Act, 1858, and to make further Provision for the Local Government of Towns and Populous Districts. 1858.

An Act for vesting in the Privy Council certain Powers for the Protection of the Public Health. 1858.

Bird (Peter Hinckes.) On the Nature, Causes, Statistics, and Treatment of Erysipelas. London: Churchill. 1858.

Bond (Francis T., M.D.) On the Pathology of Rheumatism. London: Longmans. 1858.

Buchanan (G., M.D.) St. Giles in 1857. Report to the District Board of Works. London: 1858.

Clendon (J. C.) On some severe forms of Disease arising from the Retention of Decayed Teeth. London: Richards. 1858.

Easton (J. A., M.D.) On the Elimination, Catalysis, and Counter-Action of Poisons. Glasgow: 1858.

Glaisher (James, F.R.S.) On the Meteorology of England during the Quarter ending June 30th, 1858. London: 1858.

Godard (Ernest M.) Etudes sur la Monorchidie, et la Cryptorchidie, chez L'Homme. Paris: Victor Masson. 1857.

Greenhow (E. Headlam, M.D.) An Inquiry into the different Proportions of Death produced by certain Diseases in different Districts of England. By E.Headlam Greenhow, M.D.: with an Introductory Report on the Preventibility of certain kinds of Premature Death, by John Simon, F.R.S. London: 1858. Blue Book.

Harvey (Alexander, M.D.) Four Letters to Sir James Clark on Administrative Reform in relation to the Medical Schools and the Examining Boards. London: Churchill. 1858.

Hewitt (Graily, M.D.) On the Vesicular Emphysema of the Lungs in Early Childhood. London: 1858.

Hillier (Thomas, M.D.) Report, Second Annual, on the Sanitary Condition of Saint Pancras during 1857. London. 1858.

Hogg (Jabez.) On the Ophthalmoscope. London: Churchill. 1858.

Inman (T., M.D.) On Counterirritants. London: 1858.

Letheby (H. L., M.B.) Report on the Sanitary Condition of the City of London for the Quarter ending June 26th, 1858. London: 1858.

Lindsay (W. Lauder, M.D., F.L.S.) On the Transmission of Diseases between Man and the Lower Animals. Edinburgh: 1858.

MURCHISON (Charles, M.D.) Remarks on the Changes which are supposed to have taken place in the Type of Continued Fever. London: 1858.

NOBLE (Daniel, M.D.) The Human Mind in its Relations to the Brain and Nervous System. London: Churchill. 1858.

OBSERVATIONS on the Process Patented by Mr. Falcony for Embalming and Preserving the Deceased. London: 1858.

PEARCE (Samuel.) Report on the Sanitary History of Bethnal Green. London: 1858.

REPORT of the Annual General Meeting of the South Midland Branch of the British Medical Association. London: Richards. 1858.

REPORT of the Council of the British Meteorological Society; read at the Seventh Annual Meeting. London: 1858.

[The Report contains a very interesting memoir of Sir John Ross.]

REPORT, the Thirty-first, of the Directors of Murray's Royal Asylum for Lunatics, near Perth. Perth: 1858.

RIDGE (Benjamin, M.D.) Health and Disease; their Laws, with Plain Practical Prescriptions for the People. London: Chapman and Hall. 1858.

RIGDEN (George.) Vital Statistics of Canterbury. Canterbury: 1858.

[A series of statistical facts collected with great labour, and containing much useful information.]

ROBERTSON (W. Tindal, M.D.) Sanitary Science: its Past and Present State. London: Walton and Maberly. 1858.

SNOW (John, M.D.) On Chloroform, and other Anæsthetics: their Action and Administration. Edited, with a Memoir of the Author, by B. W. Richardson, M.D. London: Churchill. 1858.

THOMSON (Dundas, M.D.) Reports on the Health and Climate of St. Marylebone. London: 1858.

WESTALL (E.) Quarterly Mortality of Croydon. April 1st to June 31st, from 1848 to 1858 inclusive.

---

### IN EXCHANGE.

Literary Gazette.
Psychological Journal.
British Medical Journal.
Ranking and Radcliffe's Abstract of the Medical Sciences.
Chemist.
Veterinarian.
Asylum Journal of Mental Science.
Quarterly Journal of the Chemical Society.
Scottish Review.
Edinburgh Medical Journal.
Edinburgh Veterinary Review
Glasgow Medical Journal.
Dublin Hospital Gazette.
American Medical Monthly
American Journal of the Medical Sciences.
New York Journal of Medicine.
Montreal Medical Chronicle.
Charleston Medical Journal.
Gazette Médicale de Paris.
Aerzliches Intelligenz-Blatt.
Medical and Surgical Reporter.
Journal of Practical Medicine and Surgery.
Journal de Physiologie.
Gazette Médicale de l'Orient

*Is now Published, price* 16*s.*

---

### THE ASTLEY COOPER PRIZE ESSAY,

ON THE

### CAUSE OF THE

### COAGULATION OF THE BLOOD:

WITH A PRACTICAL APPENDIX.

BY

BENJAMIN W. RICHARDSON, M.D., L.R.C.P.,

PHYSICIAN TO THE ROYAL INFIRMARY FOR DISEASES OF THE CHEST.

---

### Contents:

CHAPTER I. Historical Memoranda on the supposed Causes of Coagulation of the Blood.
CHAPTER II.—Cause of the Phenomenon of Coagulation: present position of the Question.
CHAPTER III.—The Author's Personal Observations and Deductions on the Condition of the Blood in the Body under certain Physiological and Pathological States.
CHAPTER IV.—Experimental Inquiry into the Physical Agencies influencing the Coagulation of the Blood.
CHAPTER V.—Experimental Inquiry into the Chemical Agencies influencing the Coagulation of the Blood.
CHAPTER VI.—Experimental Inquiry into the Chemical Agencies influencing the Coagulation of the Blood (continued).
CHAPTER VII.—Phenomenon of the Buffy Coat.
CHAPTER VIII.—Summary of Conclusions and Propositions.

APPENDIX.

1.—Superalkaline Conditions of the Blood in Relation to Disease.
2.—Ammonia as an Exhalation from the Body.
3.—Experiments, shewing the Effect of Lactic Acid on Animal Bodies (with Coloured Plates, shewing the stages of Endocarditis).
4.—Deposition of Fibrin in the Heart and Blood-vessels during Life.
5.—The Alkalies and Acids as Remedial Agents.
6.—Application of Ammonia to the Experiment of Transfusion of Blood.
7.—On some Conditions of the Blood after Death, in relation to Medico-Legal Inquiries.
8.—List of Authors.

---

*The Essay is Illustrated with numerous Wood Engravings, and with Coloured Plates by Tuffen West.*

---

"We shall merely give an outline of what we may justly call Dr. Richardson's great discovery, feeling certain that all who desire to keep themselves up to the professional science of the day will study the work for themselves....It is with extreme pride and satisfaction that we congratulate our countryman upon the appearance of his great work."—*Medical Times and Gazette.*

"We recommend to our readers a careful perusal of the whole treatise, which we look upon as not less remarkable for its high value as a model of logical research and a study for experimental physiologists, than it is memorable in the annals of our scientific literature for its luminous truths and satisfactory results."—*The Lancet.*

"The essay of Dr. Richardson affords an instructive lesson, as well as encouragement, to all who are engaged in investigating the phenomena of nature."—*British Medical Journal.*

"Nous proclamons sans hésiter que ce livre est destiné à prendre place parmi les œuvres scientifiques les plus remarquables de notre époque."—*Journal de la Physiologie.*

London: JOHN CHURCHILL, New Burlington Street.

